# Author Correction: Graphene Plasmonic Fractal Metamaterials for Broadband Photodetectors

**DOI:** 10.1038/s41598-020-69045-4

**Published:** 2020-07-16

**Authors:** Francesco De Nicola, Nikhil Santh Puthiya Purayil, Vaidotas Miŝeikis, Davide Spirito, Andrea Tomadin, Camilla Coletti, Marco Polini, Roman Krahne, Vittorio Pellegrini

**Affiliations:** 1 Graphene Labs, Istituto Italiano di Tecnologia, Via Morego 30, 16163 Genova, Italy; 2 Physics Department, Universitá degli studi di Genova, Via Dodecaneso 33, 16146 Genova, Italy; 3 CNI@NEST, Istituto Italiano di Tecnologia, Piazza San Silvestro 12, 56127 Pisa, Italy; 4 Nanochemistry Department, Istituto Italiano di Tecnologia, Via Morego 30, 16163 Genova, Italy; 5 Physics Department, Universitá di Pisa, Largo Bruno Pontecorvo 3, 56127 Pisa, Italy

Correction to: *Scientific Reports* https://doi.org/10.1038/s41598-020-63099-0, published online 23 April 2020

The Supplementary Information file that accompanies this Article contains a typographical error in the second line of Equation 5 where

$${\left(C/A\right)}^{2}$$

should read:

$${\left(A/C\right)}^{2}$$

The correct version of Equation 5 is below:

$$e=\left\{\begin{array}{c}\sqrt{1-{\left(C/A\right)}^{2},}\\ \sqrt{1-{\left(A/C\right)}^{2}},\end{array}\right. \genfrac{}{}{0pt}{}{{\text{for }} A>C,}{\text{for }C>A.}$$

This correction does not affect the conclusions of the Article.

