# Supplementary material for: Graphene Plasmonic Fractal Metamaterials for Broadband Photodetectors
Source: Sci Rep. 2020 Apr 23;10:6882. doi: 10.1038/s41598-020-63099-0 (PMC7181626; doi:10.1038/s41598-020-63099-0)
Supplement: Supplementary file 1 — Supplementary Information. [file 41598_2020_63099_MOESM1_ESM.pdf]

Supplementary Information to Graphene Plasmonic Fractal Metamaterials for Broadband Photodetectors

Francesco De Nicola,[1,*] Nikhil Santh Puthiya Purayil,[1,2] Vaidotas Miŝeikis,[3] Davide Spirito,[4] Andrea Tomadin,[5] Camilla Coletti,[3] Marco Polini,[1] Roman Krahne,[4] and Vittorio Pellegrini[1]

[1]*Graphene Labs, Istituto Italiano di Tecnologia, Via Morego 30, 16163 Genova, Italy*

[2]*Physics Department, Universitá degli studi di Genova, Via Dodecaneso 33, 16146 Genova, Italy*

[3]*CNI@NEST, Istituto Italiano di Tecnologia, Piazza San Silvestro 12, 56127 Pisa, Italy*

[4]*Nanochemistry Department, Istituto Italiano di Tecnologia, Via Morego 30, 16163 Genova, Italy*

[5]*Physics Department, Universitá di Pisa, Largo Bruno Pontecorvo 3, 56127 Pisa, Italy*

1. EXPERIMENTAL SIERPINSKI CARPETS

The main properties of the experimental Au/G Sierpinski carpets (SC) shown in Figure 1 are summarized as follows. The size of the sub-cells at a fractal order t is $L_t = L_0 3^{-t}$, where $L_0 = 10$ μm is the size of the initial cell. At each iteration the side of the sub-cells is reduced by a factor $\mathcal{L} = 3$. Since the number of empty sub-cells in the SC increases by a factor $\mathcal{N} = 8$ at every iteration, the fractal dimension is $d_H = \log \mathcal{N} / \log \mathcal{L} \approx 1.89$. The total area of the fractal is $\mathcal{A}_t = \sum_t N_t A_t$, with $N_t = 8^{t-1}$ and $A_t = L_t^2$ the number and area of the square elements, respectively, while the device channel spatial fill factor is $f = 1 - \mathcal{A}_t/(4L_0)^2$. In Table SI, the experimental main quantities associated to the five orders of the Au/G SC are reported.

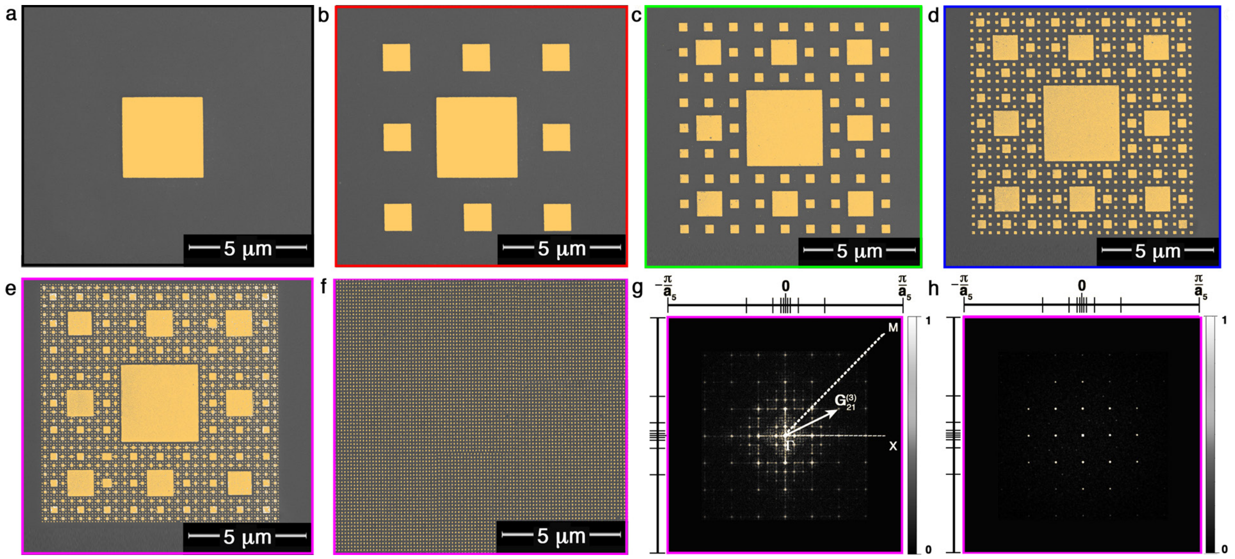

FIG. 1. **a-e**, Color-coded scanning electron microscopy (SEM) micrographs of 35 ± 3 nm thick Au SCs deposited on a G/SiO_2/Si substrate for fractal orders $t = 1$ (black), $t = 2$ (red), $t = 3$ (green), $t = 4$ (blue), and $t = 5$ (magenta). **f**, Micrograph of 35 ± 3 nm thick Au periodic array deposited on a G/SiO_2/Si with square size $L_5 = 41 \pm 3$ nm (magenta), as a comparison. Fast Fourier transform of the SEM micrographs for the SC at $t = 5$ (**g**) and the periodic array (**h**).

t	$\mathcal{N}_t$	N_t	L_t (μm)	A_t (μm^2)	$\mathcal{A}_t$ (μm^2)	f
1	8	1	3.382 ± 0.050	11.438 ± 0.338	11.438 ± 0.338	0.993
2	64	8	1.121 ± 0.011	1.257 ± 0.025	21.494 ± 0.200	0.986
3	512	64	0.390 ± 0.017	0.152 ± 0.013	31.222 ± 0.832	0.980
4	4096	512	0.130 ± 0.007	0.017 ± 0.002	39.926 ± 1.024	0.975
5	32768	4096	0.044 ± 0.003	0.002 ± 0.001	48.118 ± 4.096	0.969

TABLE I. Summary of the Au/G SC main experimental quantities.

2. REFLECTANCE SPECTRA OF AU/G SIERPINSKI CARPETS

In Figure 2 (a, b), the experimental reflectance spectra of 35 ± 3 nm thick Au/G SCs for t = 0-5 orders are shown. Localized surface plasmon (LSP) modes progressively appear in the spectrum by increasing the fractal order.

The in-plane coupling between the LSP modes of the Au squares in an Au SC and the out-of-plane coupling between the Au SC modes and graphene are compared in Figure 3. Since the exponential fitting decay constant $\ell = 396$ nm for the in-plane coupling is about two times larger than $\ell = 213$ nm for the out-of-plane coupling, the latter coupling is two times stronger than the former.

At first approximation, the position in photon energy of the LSP resonances in the reflectance spectra can be determined for small LSP wavevectors k_p by

$$\omega_n = 2\pi c/3a_n, \text{ with } n = 1, 2, \dots t, \tag{1}$$

while for large wavevectors k_p the modes tends to

$$\omega_n \equiv \omega_{sp}/\sqrt{3} = 2.40 \text{ eV}, \tag{2}$$

with $\omega_{sp} = 4.17$ eV the Au surface plasmon[1]. Better results can be achieved by the Mie-Gans model[2] for oblate metal particles in air ($\varepsilon_m = 1$) with axis $i = A, B, C$, being $A = B = L_t$ and $C = 45$ nm the Au/Ti square total thickness, which reads

$$\omega_n^i = \frac{\omega_{sp}}{\sqrt{1 + \frac{1-P_i}{P_i}\varepsilon_m}}. \tag{3}$$

The longitudinal modes $\omega_n^{A,B}$ are given by

$$P_A = P_B = \frac{1-e^2}{e^2}\left[\frac{1}{2e}\ln\left(\frac{1+e}{1-e}\right) - 1\right], \tag{4}$$

with the particle eccentricy

$$e = \begin{cases} \sqrt{1-(C/A)^2}, & \text{for } A > C, \\ \sqrt{1-(C/A)^2}, & \text{for } C > A. \end{cases} \tag{5}$$

The transversal modes ω_n^C are given by

$$P_C = \frac{1-P_A}{2}. \tag{6}$$

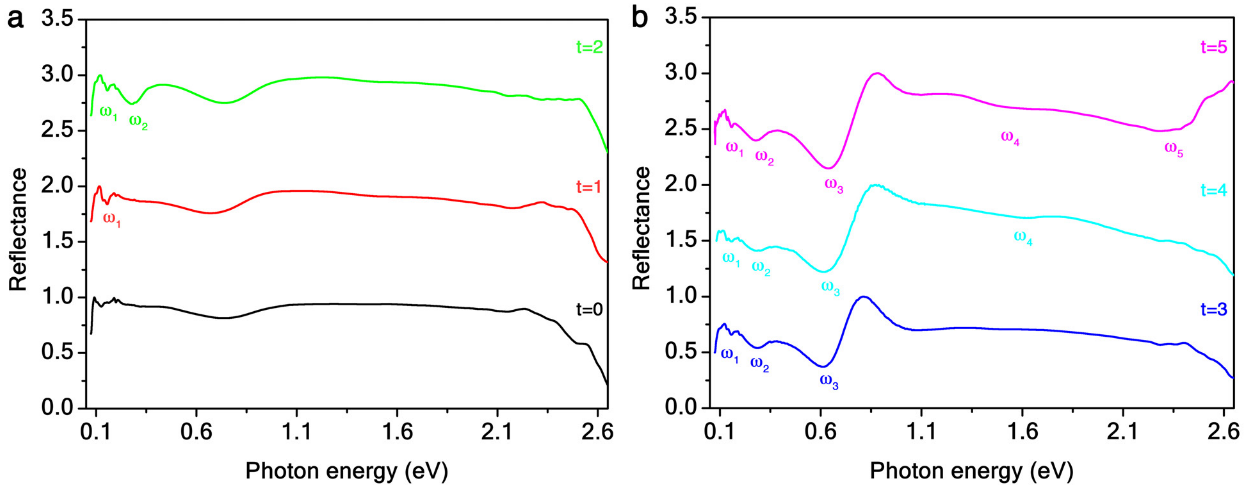

FIG. 2. Experimental reflectance spectra of Au/G SCs for t = 0-2 orders (**a**) and t = 3-5 orders (**b**). Localized surface plasmon modes ω_n are marked.

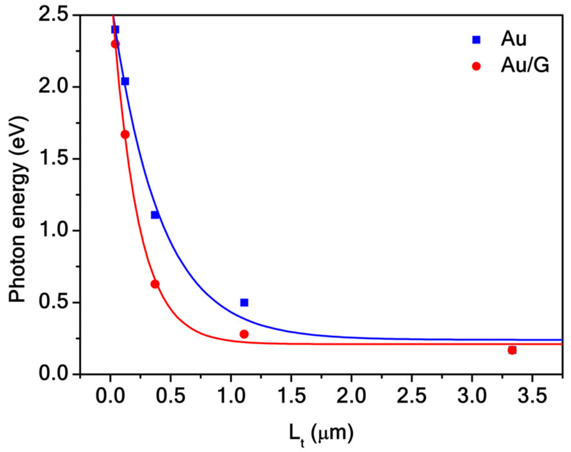

FIG. 3. Localized surface plasmon modes ω_n for an Au SC (blue squares) and an Au/G SC (red dots) for $t = 5$ as a function of the Au square size L_t. Solid lines represent exponential fits $\propto e^{-L_t/\ell}$ with decay constants $\ell = 396$ nm (blue solid line) and $\ell = 213$ nm (red solid line).

3. ELECTROMAGNETIC SIMULATION OF THE AU/G FRACTALS

In order to evaluate the electromagnetic near-field distributions of the Au/G SCs, we employed CST Microwave Studio software for finite elements method simulations. For our study we considered Au SCs deposited on $G/SiO_2/Si$ substrates. The complex dielectric function of the Ti/Au squares constituting the fractals was described by Johnson and Christy[3], and Rakic et al.[4], while the dielectric function of Si/SiO_2 substrate was given by Palik[5] and Malitson[6]. Graphene was modeled as a two-dimensional impedance surface with complex impedance $\sigma^{-1}(\omega) = X(\omega) + iY(\omega)$, being $X(\omega)$ the resistance and $Y(\omega)$ the reactance, obtained by the graphene RPA conductivity[7] for varying values of Fermi energy E_F, which reads

$$\sigma(\omega) = \frac{2e^2 k_B T}{\pi \hbar^2} \frac{i}{\omega + i\tau^{-1}} \log\left[2\cosh\left(E_F/2k_BT\right)\right] + \frac{e^2}{4\hbar}\left[H(\omega/2) + \frac{i4\omega}{\pi}\int_0^\infty d\epsilon \frac{H(\epsilon) - H(\omega/2)}{\omega^2 - 4\epsilon^2}\right],$$

$$H(\epsilon) = \frac{\sinh\left(\hbar\epsilon/k_BT\right)}{\cosh\left(E_F/k_BT\right) + \cosh\left(\hbar\epsilon/k_BT\right)}. \tag{7}$$

From DC electronic transport measurements on unpatterned graphene with typical doping $E_F = 0.4$ eV, the carrier scattering time $\tau \equiv E_F\mu/v_F^2 e = 0.1$ ps is found. In Figure 4a the calculated graphene RPA conductivity is reported along the permittivity in Figure 4b for

varying values of Fermi energy.

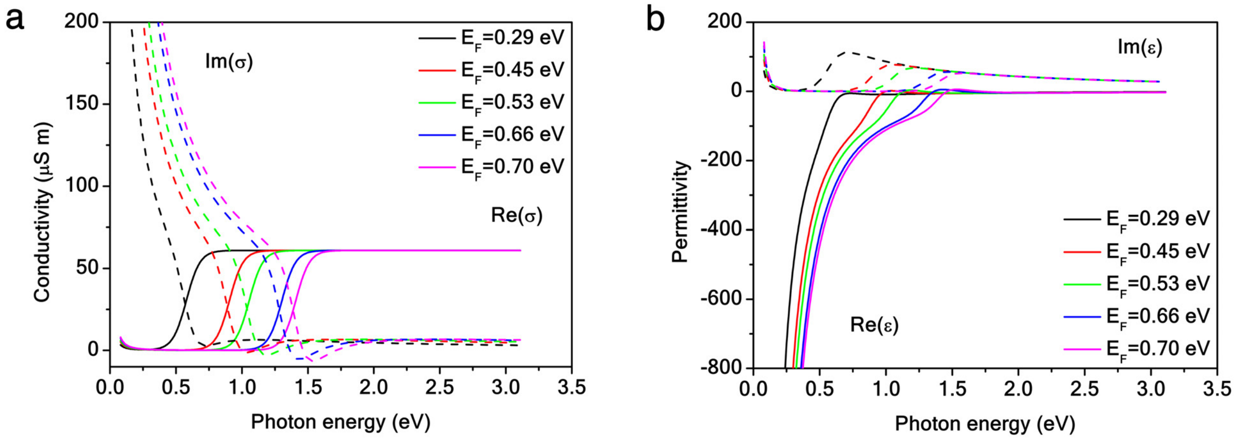

FIG. 4. Calculated graphene RPA conductivity (**a**) and permittivity (**b**) for varying values of Fermi energy with carrier scattering time $\tau = 0.1$ ps.

The design of our structures is illustrated in the inset of Figure 5. A linearly polarized plane wave (TM) radiation source with a varying wavelength is placed above the structure at normal incidence. A perfectly matched layer bounding box was implemented. The boundary conditions of the system were considered as periodic. We used a sufficiently fine mesh that gave steady and mesh-independent results for the near-field distributions. A direct solver was chosen for the solution method (MUMPS), which allowed cluster computing for parallelization.

Figure 5 shows that under quasi-static conditions ($\lambda \gg L$), the dipolar electric near-field of the central element is remarkably affected by the dipoles of the neighboring elements smaller in size, corresponding to the successive orders of the SC. Therefore, the near-field couples with elements of different size, redistributing the local scattered radiation on the overall structure, thus hierarchically localizing the electromagnetic near-field on the self-similar structures[8]. In this way, an efficient transfer of LSP excitations towards progressively smaller length scales occurs in the SC, as its order of complexity increases.

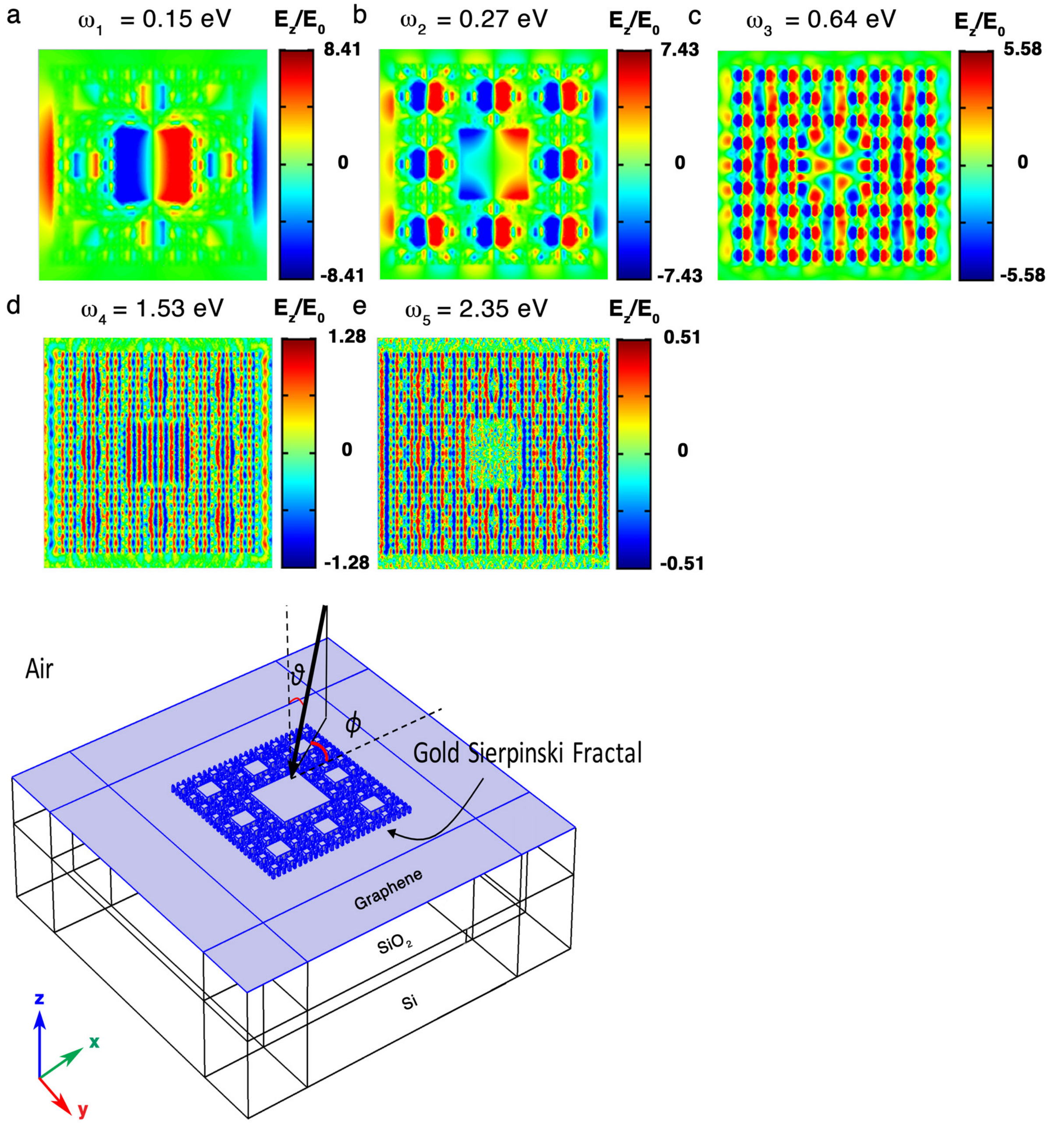

FIG. 5. Calculated electric near-field enhancement (E_z/E_0) distribution at $z = 100$ nm from an Au/G SC for $t = 5$ at their LSP resonances. The incident wave points in the $\theta = 0$ direction orthogonal to the plane of incidence with the electric field in the $\phi = 0$ direction. Inset, a sketch of the modeled Au SC deposited on a G/SiO_2/Si substrate. Figures were obtained by CST Microwave Studio software, www.cst.com

4. GRAPHENE FRACTAL METAMATERIAL CRYSTALLINITY

The crystallinity of the graphene fractal metamaterial was assessed by considering an Au/G SC (Figure 6a), a graphene SC (Figure 6b), and the original unpatterned graphene sample. In Figure 6c average Raman spectra recorded at $\lambda_{ex} = 514$ nm for the Au/G SC, the graphene SC, and for a reference unpatterned graphene are shown. It can be observed that while for the unpatterned graphene and the Au/G SC the D vibrational mode is negligible, for the graphene SC it becomes comparable to the G mode. Also, a D$'$ mode is present in the graphene SC spectrum. Both the effects can be ascribed to the reactive ion etching with oxygen plasma during the fractal patterning of graphene. In fact, plasma etching induces defects and oxygen impurities in the graphene cristalline lattice by breaking the C-C bonds and forming C-O bonds[9]. Figure 6d illustrates a strain-doping diagram[10] showing that the Au/G SC is less strained but more hole doped than the original unpatterned graphene due to the Au charge transfer, while the graphene SC is more strained than the unpatterned graphene owing to the etching process. Therefore, patterning graphene with Au fractals does not alter its crystallinity, while directly pattern graphene does.

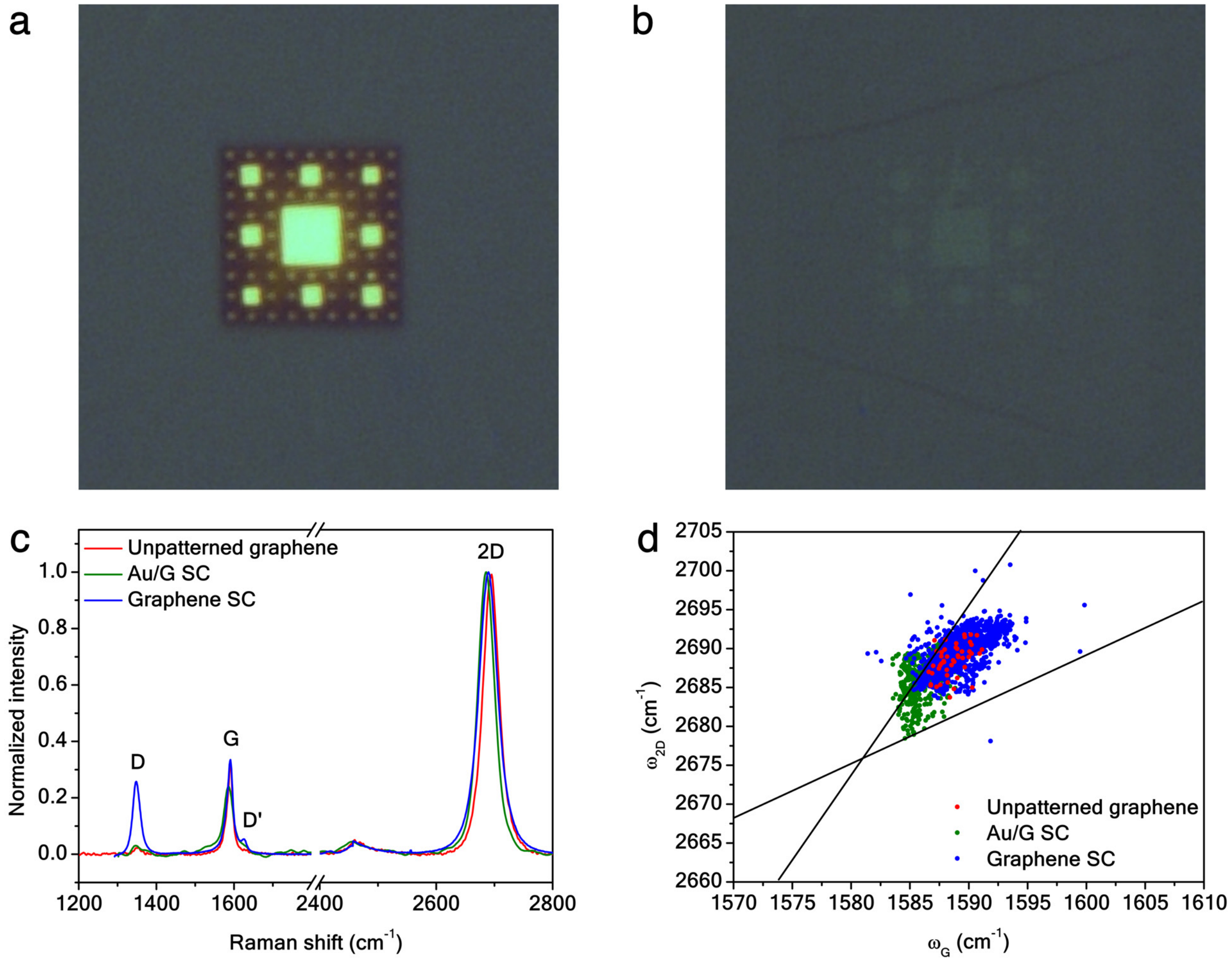

FIG. 6. Optical microscopy micrographs of an Au/G SC (**a**) and a graphene SC (**b**) for $t = 5$. **c**, Averaged Raman spectra recorded at $\lambda_{ex} = 514$ nm for the Au/G SC (green curve) in **a**, the graphene SC (blue curve) in **b**, and a reference unpatterned graphene (red curve). **d**, Strain-doping diagram for the Au/G SC (green dots) in **a** and the graphene SC (blue dots) in **b**, and for a reference unpatterned graphene (red dots). The intersection point between the upper strain line of slope 2.2 ± 0.2 and lower doping line of slope 0.70 ± 0.05 correspond to the unstrained and undoped graphene sample in Ref. 10.

5. SURFACE ENHANCED RAMAN SPECTROSCOPY

The surface enhanced Raman spectroscopy (SERS) enhancement factor was calculated for orders t = 1-5 of the SC by the relation

$$EF_{sers} = \frac{\langle I_{sample}\rangle}{\langle I_{Au}\rangle}\frac{A_{Au}}{A_{sample}N_{sample}}, \tag{8}$$

where $\langle I_{sample}\rangle$ is the average Raman intensity in adu units (1 adu = 1 count/mWs) at the selected vibrational band $\omega^\star$ measured over the SC maps, $A_{Au} = 0.785\ \mu m^2$ is the area of the laser beam spot on the reference Au film, $A_{sample} = L_t^2$ is the area of the smallest square of the fractal at order t for which the Raman enhancement is maximum, N_{sample} is the number of the smallest squares within the beam spot area, and $\langle I_{Au}\rangle$ is the average Raman intensity in adu units at $\omega^\star$ measured over the map of the ideally smooth reference Au film. Notably, electromagnetic simulations showed that the reference Au film surface roughness ($\approx$ 3 nm) provides itself an electric field enhancement factor of about 2, therefore a Raman intensity factor $\langle I_{ref}\rangle/\langle I_{Au}\rangle \approx 16$ with respect to an ideally smooth Au surface. It is worth noting that all the experimental conditions for the sample and the reference are exactly the same.

Furthermore, in order to map the SERS enhancement all over the fractal surface, we deposited on the $t = 5$ Au/G SC a thin layer of Brilliant Cresyl Blue (BCB) dye as a probe. In Figure 7 average Raman spectra recorded at $\lambda_{ex} = 532$ nm for an Au/G SC at $t = 5$ and an unpatterned graphene are shown. The resonant SERS enhancement factor calculated at the BCB vibrational band $\omega^\star = 1655\ cm^{-1}$, corresponding to the coupling of the NH_2 scissor mode with the asymmetric stretch mode of C rings[11], is 10^3.

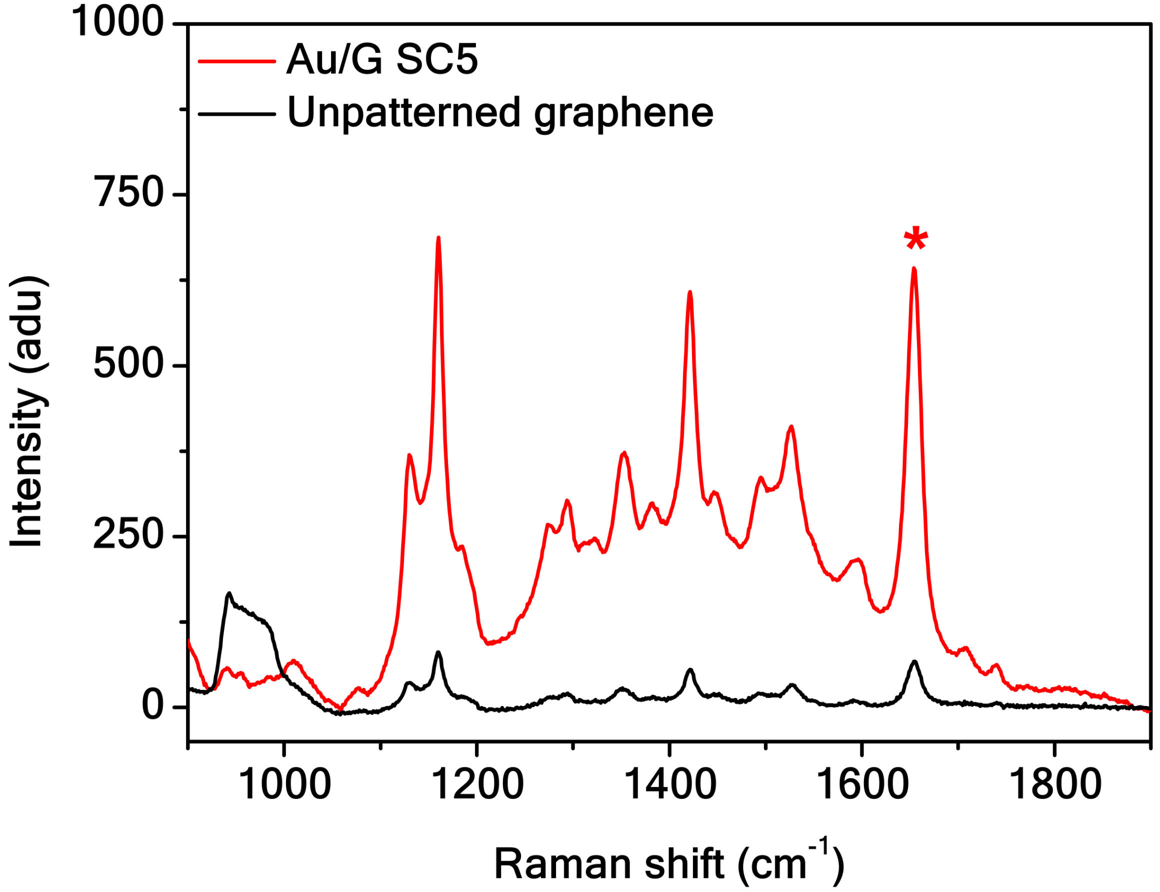

FIG. 7. Averaged Raman spectra of Au/G fractals at $\lambda_{ex} = 532$ nm for $t = 5$ and for a reference unpatterned graphene. The BCB vibrational band $\omega^{\star} = 1655$ cm^{-1} is marked.

6. AU/G SIERPINSKI CARPET PHOTODETECTORS

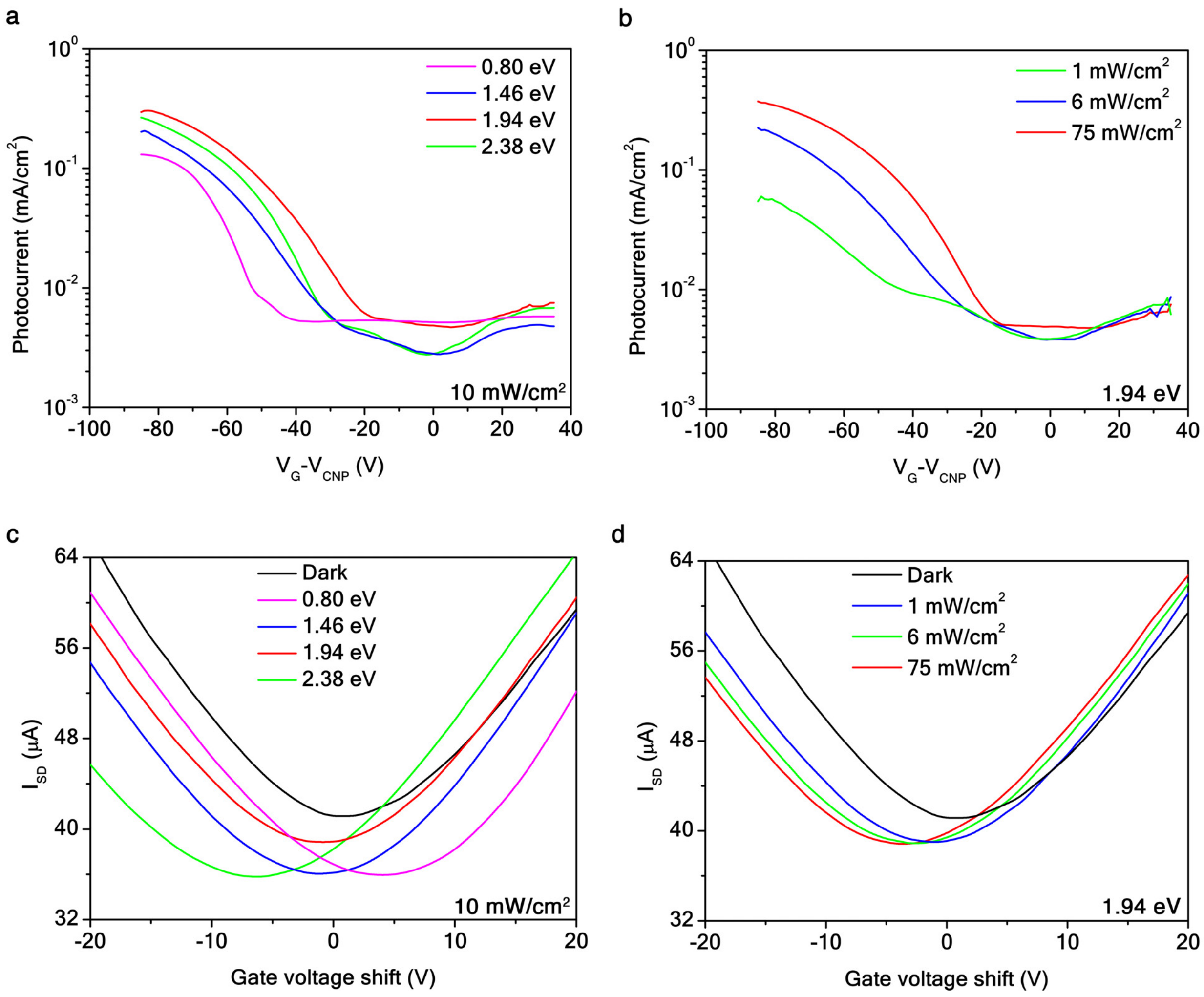

FIG. 8. AC photocurrent at $V_{SD} = 0.1$ V as a function of the relative gate voltage to the CNP for different incident laser energy at 10 mW/cm^2 (**a**) and for different powers of a 1.94 eV laser (**b**). DC current at $V_{SD} = 0.1$ V in dark and under incident light as a function of the gate voltage shift with respect to the CNP in dark and under different incident laser energies at 10 mW/cm^2 (**c**) and for different power of a 1.94 eV laser (**d**).

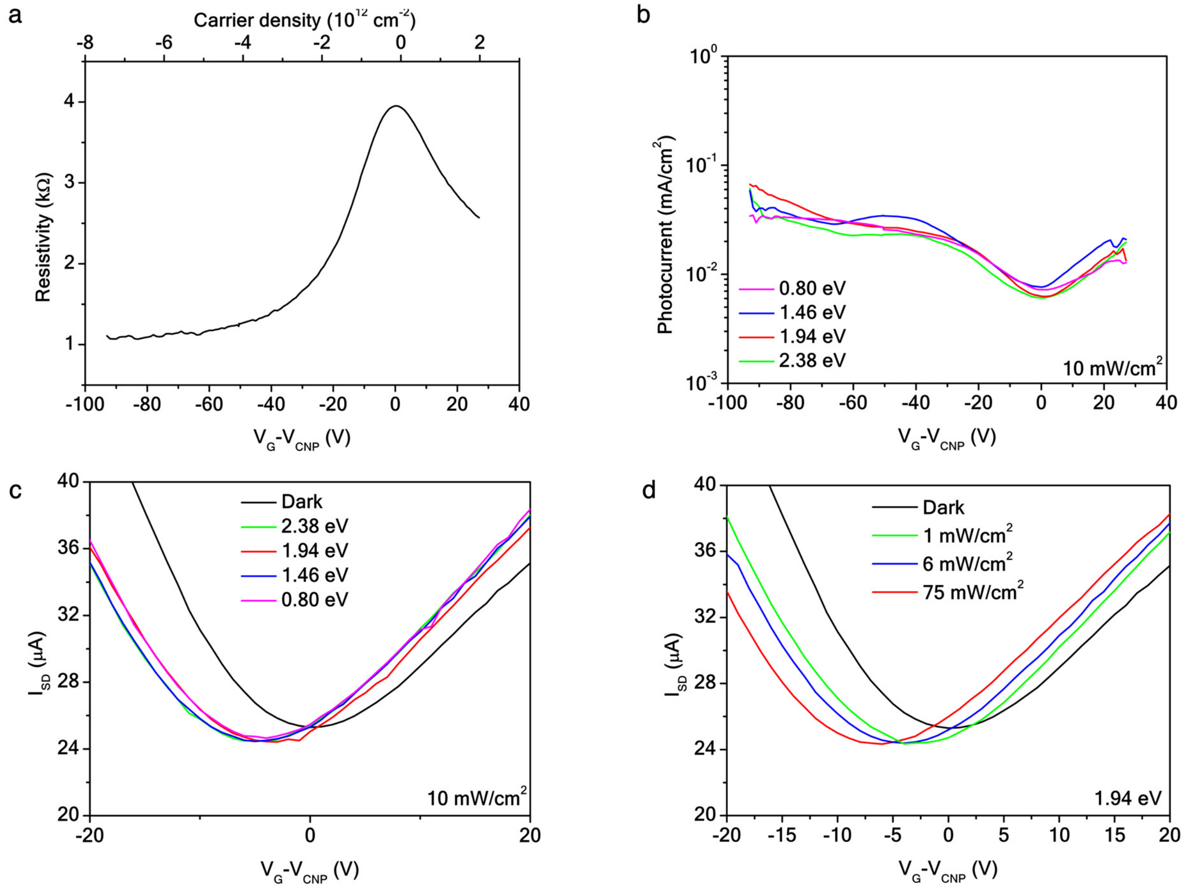

FIG. 9. **a**, Resistivity at $V_{SD} = 0.1$ V of an unpatterned graphene device as a function of the gate voltage relative to the CNP and carrier density. **b**, AC photocurrent at $V_{SD} = 0.1$ V as a function of the gate voltage relative to the CNP for different incident laser energies at 10 mW/cm^2. DC current at $V_{SD} = 0.1$ V in dark and under incident light as a function of the gate voltage shift with respect to the CNP in dark and under different incident laser energies at 10 mW/cm^2 (**c**) and for different powers of a 1.94 eV laser (**d**).

In Figure 10a, the experimental DC photovoltage $\Delta u = (I_{light} - I_{dark})\sigma^{-1}$ and the function $\Delta u^* = -\sigma^{-1} d\sigma/dV_G$ are plotted as a function of the applied gate voltage for different laser photon energy. Depending on the latter, Δu is shifted with respect to Δu^*, thus it is non-zero at V_{CNP}, exhibiting a light-induced photogating effect. In particular, Δu^* is proportional to the Mott formula for the photothermoelectric voltage $\Delta u_{PTE} = \Delta S \Delta T$, with $S = -\pi^2 k_B^2 T (3e\sigma)^{-1} (d\sigma/dV_G)_{V_G=V_{CNT}}$ the Seebeck coefficient.

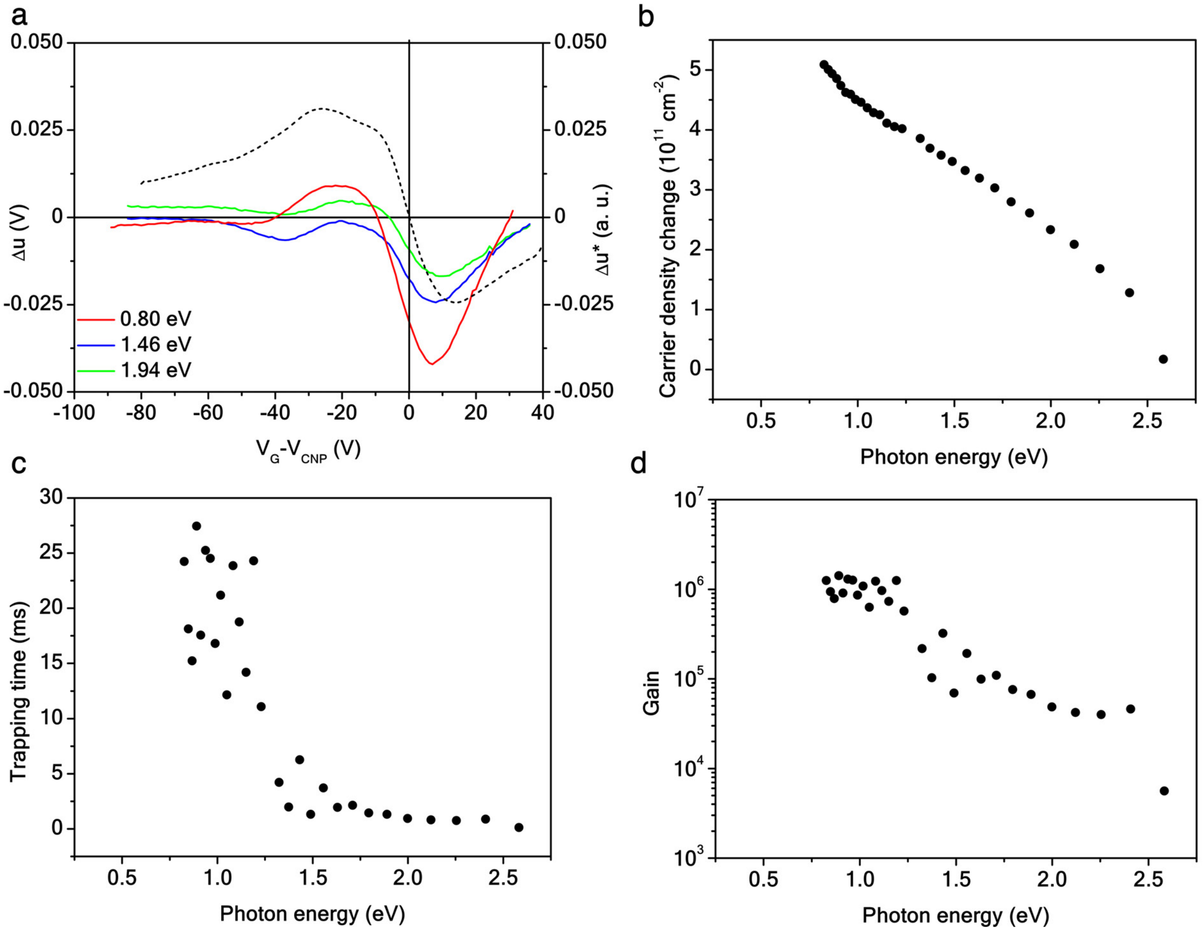

FIG. 10. **a**, Experimental DC photovoltage Δu (solid curves) and Δu^* function (dashed curve) at $V_{SD} = 0.1$ V as a function of the gate voltage relative to the CNP for different laser energy at 10 mW/cm^2. **b**, Light-induced carrier density change Δn at $V_G = -50$ V as a function of the photon energy. **c**, Electron trapping time in graphene at $V_G = -50$ V as a function of the photon energy. **d**, Photoconductive gain at $V_G = -50$ V as a function of the photon energy.

* E-mail: francesco.denicola@iit.it

[1] Maier, S. A. *Plasmonics: Fundamentals and Applications* (Springer, New York, 2007).

[2] Mayer, K. M. & Hafner, J. H. Localized surface plasmon resonance sensors. *Chem. Rev.* **111**, 3828–3857 (2011).

[3] Johnson, P. B. & Christy, R. W. Optical constants of transition metals: Ti, V, Cr, Mn, Fe, Co, Ni, and Pd. *Phys. Rev. B* **9**, 5056–5070 (1974).

[4] Rakić, A. D., Djurišic, A. B., Elazar, J. M. & Majewski, M. L. Optical properties of metallic films for vertical-cavity optoelectronic devices. *Appl. Opt.* **37**, 5271–5283 (1998).

[5] Palik, E. D. *Handbook of Optical Constants of Solids* (Academic Press, Boston, 1985).

[6] Malitson, I. H. A redetermination of some optical properties of calcium fluoride. *Appl. Opt.* **2**, 1103–1107 (1963).

[7] Koppens, F. H. L., Chang, D. E. & de Abajo, F. J. G. Graphene plasmonics: A platform for strong light-matter interactions. *Nano Lett.* **11**, 3370–3377 (2011).

[8] De Nicola, F. et al. Multiband plasmonic Sierpinski carpet fractal antennas. *ACS Photonics* **5**, 2418–2425 (2018).

[9] Ferrari, A. C. & Basko, D. M. Raman spectroscopy as a versatile tool for studying the properties of graphene. *Nat. Nanotechnol.* **8**, 238–246 (2013).

[10] Lee, J. E., Ahn, G., Shim, J., Lee, Y. S. & Ryu, S. Optical separation of mechanical strain from charge doping in graphene. *Nat. Commun.* **3**, 1–8 (2012).

[11] Nieckarz, R. J., Oomens, J., Berden, G., Sagulenkoa, P. & Zenobi, R. Infrared multiple photon dissociation (IRMPD) spectroscopy of oxazine dyes. *Phys. Chem. Chem. Phys.* **15**, 5049–5056 (2013).
